# Microbiota profiling from biopsied tissues in complex infections: a diagnostic and prognostic analysis through metagenomic next-generation sequencing

**DOI:** 10.3389/fcimb.2025.1567981

**Published:** 2025-05-08

**Authors:** Tiange Song, Lin Yin, Xiaoli Zhou, Xiaoyan Tao, Dandan Tie, Jie Zhang, Li Jiang

**Affiliations:** ^1^ Department of Laboratory Medicine, Sichuan Provincial People’s Hospital, School of Medicine, University of Electronic Science and Technology of China, Chengdu, Sichuan, China; ^2^ Sichuan Provincial Key Laboratory for Human Disease Gene Study, Sichuan Provincial People’s Hospital, University of Electronic Science and Technology of China, Chengdu, Sichuan, China; ^3^ Research Unit for Blindness Prevention of Chinese Academy of Medical Sciences (2019RU026), Sichuan Academy of Medical Sciences, Chengdu, Sichuan, China

**Keywords:** complex infections, tissue biopsy, metagenomic next-generation sequencing, diagnostic, prognostic

## Abstract

**Background:**

Infectious diseases that require tissue biopsy are usually more difficult to diagnose through conventional microbiological tests (CMT), and knowledge of the infection microbiota pattern from biopsied tissues remains incomplete. Our study aimed to investigate the diagnostic and prognostic value of metagenomic next-generation sequencing (mNGS), characterize the microbiota profile from biopsied tissues, and examine its relationship with clinical outcomes.

**Methods:**

This retrospective cohort study included 110 patients who underwent tissue biopsy and sent both mNGS and CMT due to suspected complex infection. Microbiota patterns were illustrated via unsupervised hierarchical clustering analysis. Multivariate regression analysis was used to investigate the effect measures.

**Results:**

The sensitivity of mNGS was significantly higher than that of CMT regarding bacteria (87.23% *vs* 40.43%, P=0.01), viruses (100% *vs* 5.56%, P<0.001), and fungi (87.5% *vs* 28.6%, P=0.04). Polymicrobial infection accounted for 45.2% (33/73) of the infection samples. In skeletal articular biopsied tissues, Staphylococcus presented the highest mean abundance among different species of bacteria (21.2% of all bacterial reads, standard deviation (SD) 38.9). Anaerobic bacteria (24.0%, SD 25.9) represented the most common bacteria in biopsied tissue from the lung or mediastinum. The presence of gram-negative bacteria (adjusted OR 5.21, 95% CI 1.39–19.43, P=0.01), Enterobacteriaceae (adjusted OR 5.71, 95% CI 1.17–28.03, P=0.03) and Staphylococcus (adjusted OR 8.64, 95% CI 1.95–38.34, P=0.005) was associated with an increased risk of treatment failure. Early mNGS sampling within 7 days after admission was associated with a significantly decreased risk of all-cause mortality (HR 0.18, 95% CI 0.04–0.94; P=0.04), treatment failure (OR 0.17, 95% CI 0.05–0.66; P=0.01), and increased probability of clinical resolution (OR 3.03, 95% CI 1.24–7.40; P=0.01).

**Conclusion:**

mNGS demonstrates significant diagnostic and prognostic efficacy in patients undergoing tissue biopsy for suspected complex infections. The presence of Gram-negative bacteria, Enterobacteriaceae, and Staphylococcus is associated with a higher probability of treatment failure, which underscores the advantage of using mNGS to guide more aggressive antibiotic strategies.

## Background

Tissue biopsy is a fundamentally important diagnostic tool for infections, particularly complex infections, when the pathogens are difficult to detect through other means or when the specific localization of a confined infection needs to be confirmed (Langer et al., 1994). In these clinical scenarios, broad-spectrum antibiotics are often started empirically before the identification of specific organisms that cause infection. Therefore, obtaining exact information on pathogens from limited biopsied tissues is crucial for tailoring treatment, improving therapeutic responses, and ensuring optimal patient care.

Traditionally, clinicians usually perform conventional microbiological tests (CMTs), including microbial culture, histopathology and real-time quantitative polymerase chain reaction (RT–qPCR), to detect pathogenic microorganisms. However, several limitations hinder their ability to meet the present demands for rapid and accurate infection diagnosis. With respect to the traditional gold-standard culture test, the posit ive rate can be low due to the history of antibiotic application, the presence of fastidious microorganisms, and the presence of competing microbes (Zouggari et al., 2022; Angeles-de Paz et al., 2023). The effectiveness of molecular techniques such as RT–qPCR is constrained by the genetic variability of the pathogens, and the sample size is often limited because of the low abundance of these pathogens. Therefore, given the complexity of etiology and difficulty in diagnosing infectious biopsied tissues, highly sensitive and precise diagnostic approaches are necessary.

In the past decade, metagenomic next-generation sequencing (mNGS) has become more commonly used in the clinical diagnosis of complex infectious diseases (Han et al., 2019; Qian et al., 2020; Zheng et al., 2021; Liu et al., 2022). Though it has not been considered as the first-line testing method with limited accessibility (Liu, 2024), mNGS has the following benefits. The primary advantage of mNGS is unbiased sampling, allowing for the wide-ranging detection of both recognized and unforeseen pathogens and revealing rare or novel organisms (Chien et al., 2022). Furthermore, mNGS can offer quantitative assessment of the abundance of organisms in a sample through the analysis of sequenced reads, which is valuable for identifying multiple microbial infections (Salipante et al., 2014). The diagnostic value of mNGS has been confirmed in respiratory (Zheng et al., 2021), central nervous system (Wilson et al., 2018; Piantadosi et al., 2021), blood stream (Jing et al., 2021), and other various types of infections, but evidence in biopsied tissue is lacking.

Given the small amount of biopsied tissue, potential coinfection of multiple organisms, and insensitive diagnostic test, knowledge of the landscape of microbiology in biopsied tissues from complex infections remains incomplete. The aim of our study was to investigate the diagnostic value of mNGS, characterize the microbiota profile from biopsied tissues, and evaluate the impact of mNGS timing on the overall survival, treatment failure, and clinical resolution of patients with complex infections.

## Methods

### Study design and settings

This retrospective cohort study reviewed patients who underwent tissue biopsy due to complex infection and underwent both mNGS and CMT tests at a single tertiary center from November 2021 to May 2023. The study was performed according to the Strengthening the Reporting of Observational Studies in Epidemiology (STROBE) statement (von Elm et al., 2007) and the STARD 2015 reporting guideline: an updated list of essential items for reporting diagnostic accuracy studies (Bossuyt et al., 2015) (checklist shown in **Supplementary Files 1 and 2**). Approval from the Sichuan Provincial People’s Hospital institutional review board committee was obtained for this project. Our study complied with the principles outlined in the Declaration of Helsinki, and patient informed consent was waived because of retrospective data collection.

### Participants and samples

All consecutive patients were screened from a retrospective mNGS microbial infection database at Sichuan Provincial People’s Hospital. The inclusion criteria of our study were as follows: 1) patients who underwent tissue biopsy due to suspected complex infection and 2) patients whose biopsied tissue was sent for both mNGS and CMT tests. Eligible patients were excluded if they met the following criteria: 1) incomplete clinical records, 2) unavailable mNGS results, or 3) follow-up examination after therapy.

The definition of suspected complex infection included at least one of the following conditions: 1) fever over 38°C of unknown origin or cause for more than two weeks; 2) clinical evidence of refractory confined local infection that was hard to diagnose through culture tests or did not respond to empirical antibiotics; 3) presence of multisite infection or coinfection of different types of pathogens; and 4) infection with coexisting compromised immune conditions.

Tissue biopsy was performed either by needle puncture or surgical sampling after admission. The samples were rigorously stored in aseptic containers and sent for analysis within one hour. If immediate processing is not feasible, samples can be preserved at -80°C and analysed within 12 hours.

### Conventional microbiological tests

All collected tissue samples were sent for a series of conventional microbiological laboratory tests. For bacterial and fungal detection, standard procedures predominantly involve microbiological culturing. The detection of viruses and Mycoplasma predominantly employs real-time quantitative PCR methodologies targeting various types of viruses, *Mycoplasma pneumoniae*, and Chlamydophila pneumoniae. Parasitological detection primarily employs microscopic examination. In addition, we referred to other supplementary serological assessments (G test, GM test, *M. pneumoniae* antibody assays, and *Mycobacterium tuberculosis* antibody tests) to aid in diagnosis.

### mNGS methods

For larger tissue samples, an initial homogenization step was carried out aseptically with the addition of normal saline. Subsequently, 7.2 μL of lysozyme was introduced into 0.6 mL of the tissue suspension. This mixture was then transferred to a 1.5 mL microcentrifuge tube containing 250 μL of 0.5 mm glass beads, followed by rigorous homogenization via the FastPrep-24™ 5G Homogenizer and Lysis System. After this, 0.3 mL of the sample was aliquoted into a new 1.5 mL microcentrifuge tube, and DNA extraction was performed via the TIANamp Micro DNA Kit (DP316, TIANGEN BIOTECH). The genomic DNA was then fragmented to produce 100–500 bp fragments using BGI’s proprietary Segmentase enzyme. These fragments were subsequently purified with magnetic beads to select the main fragment size range of 280–320 bp. These were then end-repaired, adenylated at the 3’ end, and ligated with adapters bearing a “T” overhang. After LM-PCR amplification and purification, the library was constructed. The library was subjected to quality control via the Agilent 2100 Bioanalyzer, and upon passing through the QC, DNA nanoballs (DNBs) were generated for sequencing on the BGISEQ-50/MGISEQ-2000 platform with a PE100 + 10 sequencing strategy.

For data processing, an initial quality assessment of the raw sequencing data was performed to eliminate low-quality reads and those contaminated with adapter sequences. Alignments to the HG19/HG20 genomes were executed via BWA software, with an evaluation of capture efficiency. Variant calling for single nucleotide variants (SNVs) and insertions and deletions (indels) was conducted via the GATK toolkit. The identified variants were then cross-referenced against several databases, including NCBI dbSNP, HapMap, the 1000 Genomes Project, and a database of 100 healthy Chinese adults, to annotate and filter the detected mutations. High-quality sequencing data were generated by removing low-quality reads, followed by computational subtraction of human host sequences mapped to the human reference genome (hg19) via Burrows–Wheeler alignment. The remaining data were classified by alignment to the Pathogens Metagenomic Database, which consists of bacteria, fungi, viruses and parasites. The classification reference databases were downloaded from NCBI (ftp://ftp.ncbi.nlm.nih.gov/genomes/). The assessment of the reference standard was based on clinical information and both CMT and mNGS test results.

### Microbiota pattern illustration

To assess the microbiota distribution across different tissue types, the species abundance was calculated via mNGS. The absolute abundance of each microorganism within a sample serves as an indicator of the microbial load, an approach inspired by the RPKM method. The formula for determining the absolute abundance of certain species is as follows: Absolute Abundance of a Species = (mapped reads number* 1Kbp * 1Mreads)/(Total Reads * genome length). The abundances of bacteria and viruses were analyzed via unsupervised hierarchical clustering, and heatmaps were generated via the Pheatmap R package.

### Data collection

The data collected for each included patient were as follows: 1) demographic data, including age, sex, major comorbidities (hypertension, diabetes, heart diseases, pulmonary diseases, chronic kidney disease, immunocompromised conditions, cancer, etc.); 2) tissue biopsy details, including biopsy samples and biopsy procedures; 3) clinical manifestations of infection, including onset, infection sites, and blood test results; and 4) diagnostic tests, including CMT results, mNGS results, the timing of mNGS sampling, and reference standard diagnosis.

### Outcomes of interest

The primary outcome of interest was 6-month overall survival. The secondary outcomes included 6-month freedom from treatment failure, the 6-month clinical resolution rate to antibiotic therapy, and the length of hospital stay. Treatment failure was defined as a composite outcome that included no clinical or microbiological cure after treatment, clinical exacerbation of symptoms, admission to the intensive care unit, the need for invasive intervention or surgery or infection-related death. The clinical resolution rate was based on the resolution of symptoms and signs associated with the infection, for example, no fever or normal blood test results.

### Statistical analysis

Continuous data are shown as the means ± standard deviations (SDs) or as medians with interquartile ranges when not normally distributed. Categorical data are displayed as counts and percentages. The reference standard was based on the identification of the microbiological etiology and clinical composite diagnosis. The χ2 test or Fisher’s exact test was used to compare the diagnostic performance between mNGS and CMT. Generalized linear model was used to investigate the association between early mNGS timing, microbial classification and length of stay.

Long-term rates are presented as the means of proportions with 95% confidence intervals (CIs). Kaplan–Meier curves and log-rank tests were used to compare time–to–event outcomes. Multivariate logistic regression and Cox proportional hazard regression were applied to calculate adjusted odds ratios (ORs) and adjusted hazard ratios (HRs) with corresponding 95% CIs for short-term and long-term outcomes. Covariates included in the multivariate analyses were identified by their clinical relevance and impact on the outcome measures in the univariate analysis. Multiple imputation was used to handle missing covariate data. All the statistical analyses were performed via R studio Version 1.2.1335 (http://www.R-project.org) and Empower (R) (www.empowerstats.com, X&Y solutions, Inc., Boston, MA).

## Results

### Characteristics of the included patients

**Figure 1:**
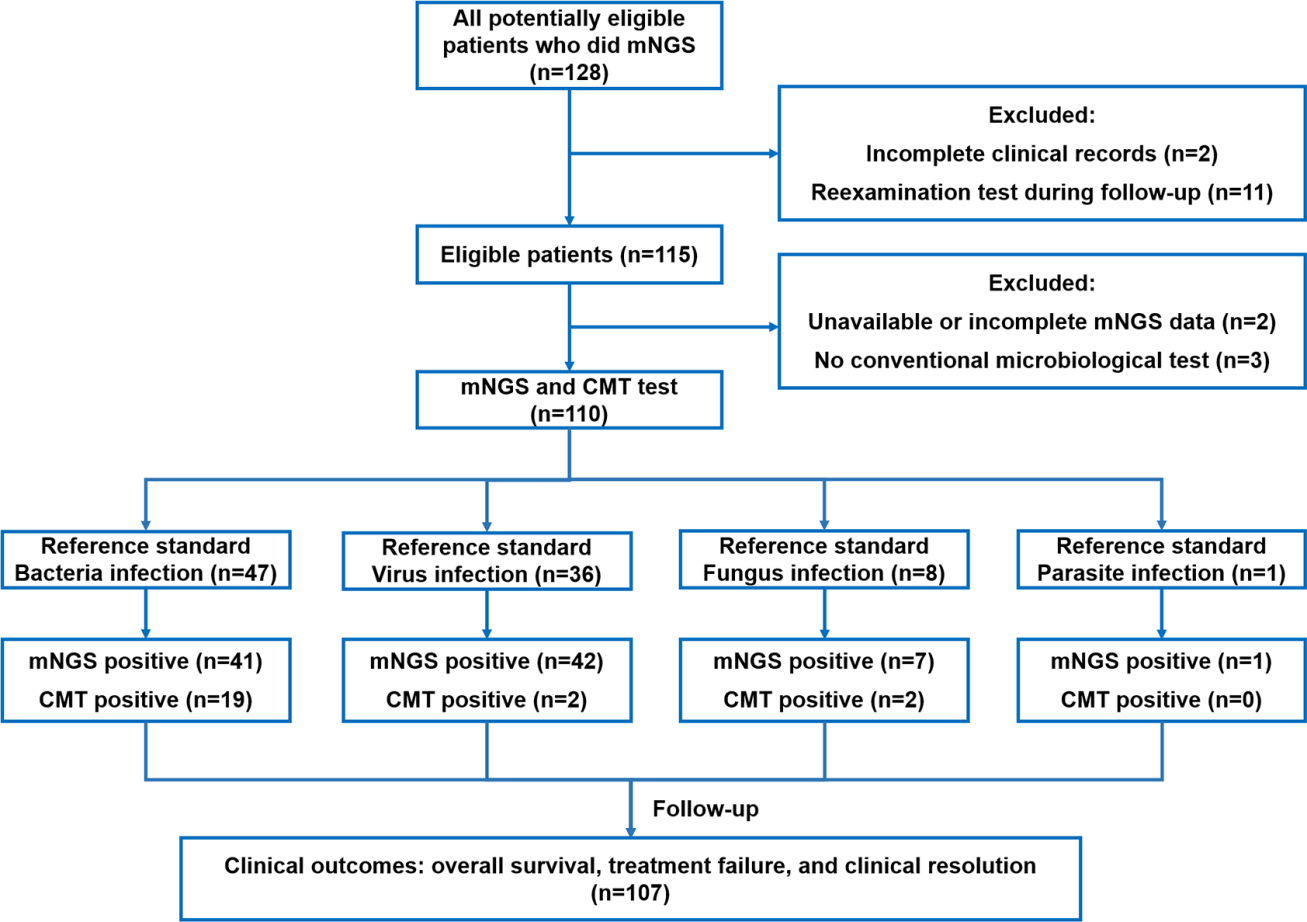
Flow diagram.

A total of 110 patients with suspected complex infection were enrolled in our study, including 49 (44.55%) females and 61 (55.45%) males. A detailed flow diagram of patient selection is shown in **Figure 1**. The mean age of the population was 49.00 ± 18.32 years. The most common comorbidity was pulmonary disease (n=30, 27.27%), followed by hypertension (n=17, 15.45%), connective tissue disease (n=17, 15.45%), heart disease (n=14, 12.73%), diabetes (n=13, 11.82%), chronic kidney disease (n=12, 10.91%), and cancer (n=12, 10.91%). Immunocompromised patients accounted for 3.64% of the total population. Nearly half of the patients (n=45, 40.91%) had a continuous intermittent fever over 38.5°C. The results of the blood tests and a summary of the baseline characteristics are shown in **Table 1**.

**Table 1:** Baseline characteristics of included patients.

Demographics	Data*
**Age-years**	49.00 ± 18.32
**Onset**	38.0 (20.0-130.0)
Gender	
** Female**	49 (44.55%)
** Male**	61 (55.45%)
**Hypertension**	17 (15.45%)
**Diabetes**	13 (11.82%)
**Cardiovascular disease**	14 (12.73%)
**Pulmonary diseases**	30 (27.27%)
**Chronic kidney diseases stage 4-5**	12 (10.91%)
**Immunosuppresion**	4 (3.64%)
**Connective tissue disease**	17 (15.45%)
**Cancer**	12 (10.91%)
**Persistent fever over 38.5**	45 (40.91%)
**Injury**	4 (3.64%)
**WBC-10^9^/L**	8.46 ± 5.61
**NEUT%**	66.58 ± 22.54
**EOS%**	1.04 ± 1.63
**LYMPH%**	17.86 ± 10.70
**CRP-mg/dl**	47.41 ± 46.03
**PCT-ng/ml**	0.42 ± 0.85
**IL6-pg/ml**	101.43 ± 158.09

*Continuous data are presented as mean ± stand deviation (SD) or median (interquartile range). WBC, white blood cell; NEUT, neutrophil; EOS, eosinophil; LYMPH, lymphocyte; CRP, C-reactive protein; PCT, procalcitonin; IL6, Interleukin 6.

### Diagnostic performance of mNGS and CMT

**Figure 2:**
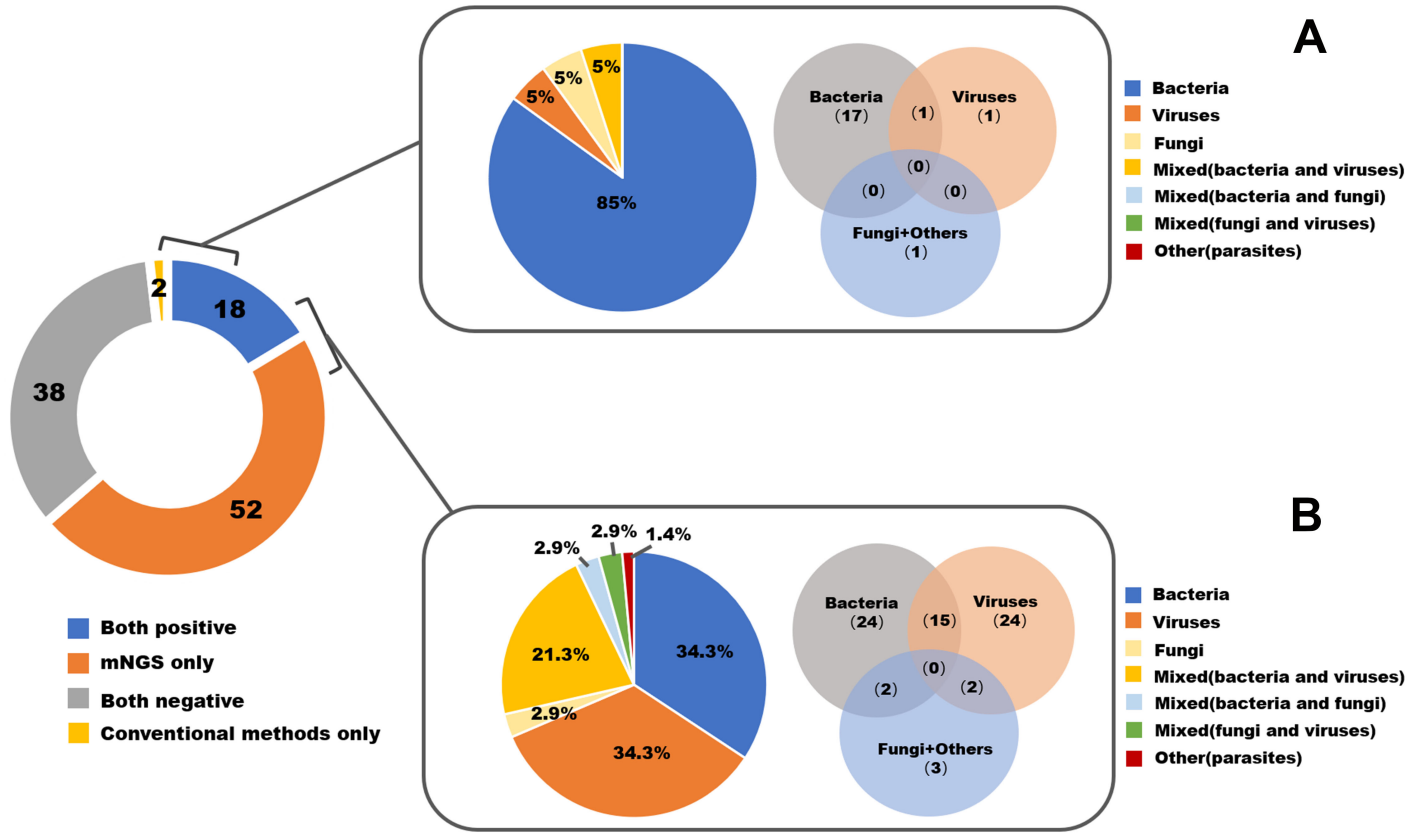
The pathogen spectrum by mNGS and conventional methods in all 110 enrolled patients. **(A)** Pathogen profiles detected by conventional methods. **(B)** Pathogen profiles detected by mNGS.

The comparison of diagnostic values between mNGS and CMT is summarized in **Table 2**. For each type of pathogen, the sensitivity of mNGS was significantly greater than that of CMT with respect to bacteria (87.23% *vs* 40.43%, P=0.01), viruses (100% *vs* 5.56%, P<0.001), and fungi (87.5% *vs* 28.6%, P=0.04). Parasites were detected in only one sample by mNGS and were not captured by CMT. The specificities of mNGS and CMT were similar and nearly reached 100% for bacteria, viruses and fungi. In terms of types of biopsied tissue, mNGS presented significantly greater sensitivity compared to CMT in the majority of types of tissues, including brain (100% *vs* 16.67%, P=0.02), eye or nose (100% *vs* 28.57%, P=0.02), musculoskeletal (88.23% *vs* 41.17%, P=0.01), bone marrow (100% *vs* 20%, P<0.001), skin and subcutaneous (100% *vs* 16.67%, P=0.02) tissues. For lung or mediastinum tissues (91.67% *vs* 58.33%, P=0.15) and liver or kidney tissues (100% *vs* 0%, P=0.10), the sensitivity of mNGS was greater but did not reach statistical significance. The pathogen spectra detected by mNGS and conventional methods are displayed in **Figure 2**.

**Table 2:** Diagnostic performance of mNGS and CMTs in different types of pathogens and biopsy specimens.

	mNGS					CMT				
	Sensitivity	Specificity	PPV	NPV	Accuracy	Sensitivity	Specificity	PPV	NPV	Accuracy
Types of pathogen										
**Bacteria**	87.23	100	100	91.3	94.54	40.42	100	100	69.23	74.54
**Virus**	100	91.89	85.71	100	94.54	5.55	100	100	68.51	69.09
**Fungas**	87.5	100	100	99.02	99.09	28.57	100	100	95.37	95.45
**Parasite**	100	100	100	100	100	0	100	0	99.09	99.09
Types of biopsied tissue										
**Brain**	100	100	100	100	100	16.66	100	100	28.57	37.5
**Eye/nose**	100	100	100	100	100	28.57	100	100	28.57	44.44
**Vertebrae/spinal cord/joint**	88.23	100	100	60	90	41.17	100	100	23.07	50
**Bone marrow**	100	86.66	90.90	100	94.28	20	100	100	48.38	54.28
**Lung/mediastinum**	91.66	100	100	85.71	94.44	58.33	100	100	54.54	72.22
**Liver/Kidney**	100	100	100	100	100	0	100	100	50	50
**skin/subcutaneous/lymph node**	100	100	100	100	100	16.66	100	100	54.54	58.33
**semen**	100	100	100	100	100	0	100	100	0	0

Data are present as percentages. PPV, Positive Predictive Value; NPV, Negative predictive value.

### Microbiota profiling and abundance

**Figure 3:**
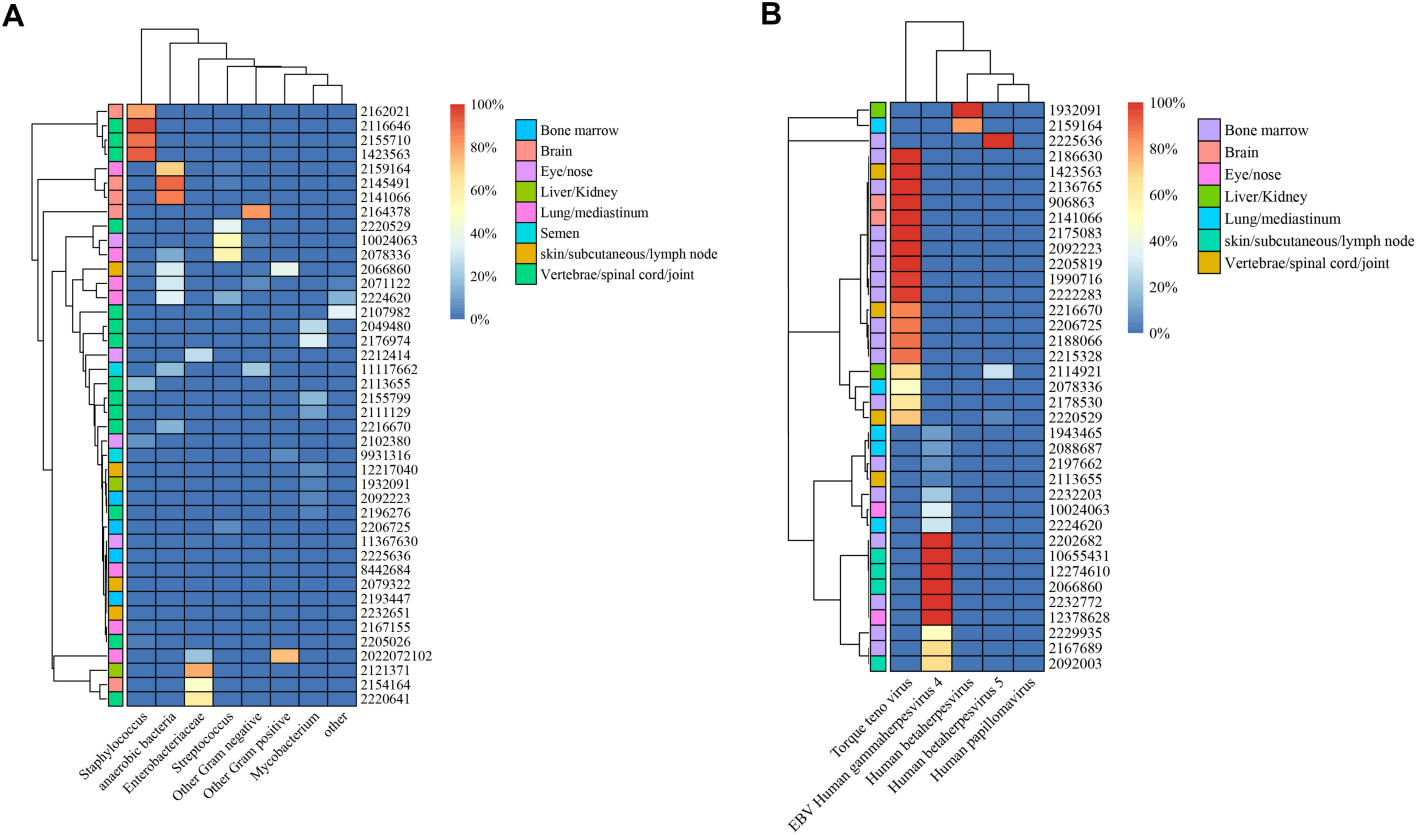
Illustration of microbiota patterns from biopsied tissues in complex infections. Heatmap of unsupervised hierarchical clustering in bacterial **(A)** and viral **(B)** infections.

In total, we identified 42 different bacterial species, five virus species, six fungal species, and one type of parasite. The distributions and classifications of all types of pathogens are shown in **Supplementary File 3**. The identified bacteria were classified into eight groups: anaerobic (9 samples), Enterobacteriaceae (7 samples), Staphylococcus (9 samples), Streptococcus (8 samples), Mycobacterium (9 samples), other gram-positive bacteria (3 samples), other gram-negative bacteria (7 samples), and other bacteria (3 samples). Nearly half (33/73, 45.2%) of the biopsied infection samples were polymicrobial (details are shown in **Supplementary File 4**). In the skeletal articular system involving the vertebrae, spinal cord and joints, Staphylococcus presented the highest mean abundance among the different bacterial species (21.2% of all bacterial reads, standard deviation (SD) 38.9), followed by Mycobacterium (6.3%, SD 10.8). For biopsied tissue from the lung or mediastinum, anaerobic bacteria (24.0%, SD 25.9) and Streptococcus (10.4%, SD 21.0) represented the most common bacteria. The abundances of bacteria from different types of biopsied tissues are shown in **Figure 3A**.

With respect to the abundance of viruses, the torque teno virus dominated in the bone marrow (56.8%, SD 47.4), brain (100%, SD 0), and skeletal articular system (62.2%, SD 44.3). Epstein–Barr virus (EBV) human gammaherpesvirus 4 was found to be the most prevalent virus in skin and subcutaneous tissues (91.7, SD 16.7) and eye or nose tissues (66.7, SD 47.1). A summary of the virus abundance in different types of biopsied tissues is displayed in **Figure 3B**.

### Impact of early mNGS timing on clinical outcomes

**Figure 4:**
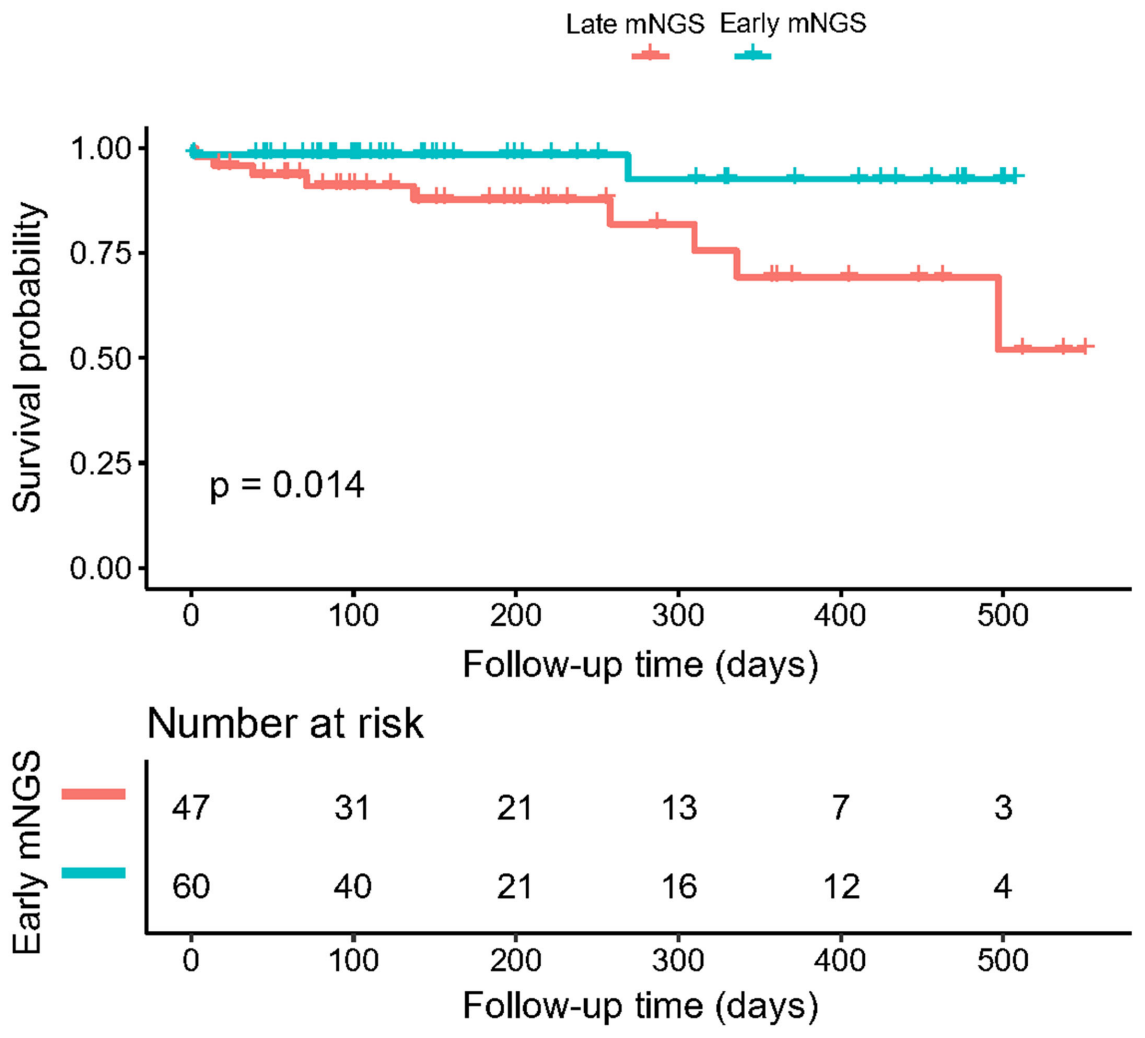
Kaplan–Meier curves illustrating the effect of early mNGS tests on the overall survival of patients with complex infections.

Among all biopsied tissues, 63 samples were sent for early mNGS within 7 days after admission. The length of stay in the early mNGS timing group was significantly shorter than that in the late mNGS timing group [12 (9–17.5) days *vs* 26 (15.5–34) days, P<0.001]. The median follow-up time of the included patients was 146.50 (78.75–315.50) days. As shown in **Figure 4**, we observed significant differences in overall survival between early and late mNGS. After adjusting for age, sex and major comorbidities, the results of multivariate regression analyses also confirmed that early mNGS timing was associated with a significantly decreased risk of all-cause mortality (HR 0.18, 95% CI 0.04–0.94, P=0.04), treatment failure (OR 0.17, 95% CI 0.05–0.66, P=0.01), and a greater probability of clinical resolution (OR 3.03, 95% CI 1.24–7.40, P=0.01). The detailed results of logistic regression analysis of length of stay, treatment failure and clinical resolution are shown in **Table 3**.

**Table 3:** Association between early mNGS timing, microbiota profile and infection-specific clinical outcomes.

	Non-adjusted		Adjust model I#		Adjust model II*	
Exposure	β/OR (95%CI)	P value	β/OR (95%CI)	P value	β/OR (95%CI)	P value
Length of stay						
**Early mNGS *vs* Late mNGS**	-13.43 (-18.14, -8.71)	<0.0001	-13.66 (-18.48, -8.84)	<0.0001	-12.76 (-17.65, -7.88)	<0.0001
Presence of the following pathogens						
***Mycobacterium tuberculosis***	-2.59 (-11.53, 6.35)	0.571	-2.11 (-11.27, 7.05)	0.653	-1.01 (-10.17, 8.16)	0.830
** Gram-negative**	8.65 (1.10, 16.20)	0.027	8.48 (0.81, 16.15)	0.033	8.81 (0.92, 16.70)	0.031
** Enterobacteriaceaeǂ**	14.68 (4.57, 24.79)	0.005	15.07 (4.74, 25.40)	0.005	15.06 (4.64, 25.49)	0.006
** Gram-positive**	-2.48 (-9.19, 4.23)	0.470	-2.91 (-9.78, 3.97)	0.410	-1.88 (-8.79, 5.02)	0.595
** *Staphylococcus aureus***	-2.12 (-10.10, 5.86)	0.604	-2.26 (-10.37, 5.84)	0.586	-1.13 (-9.26, 7.01)	0.787
** Streptococcusƚ**	-3.51 (-13.39, 6.36)	0.488	-4.33 (-14.46, 5.80)	0.404	-3.70 (-13.77, 6.36)	0.473
** Anaerobes**	2.75 (-7.13, 12.63)	0.587	2.25 (-7.89, 12.40)	0.664	2.90 (-7.16, 12.96)	0.574
** Virus**	3.59 (-2.20, 9.37)	0.227	3.35 (-2.60, 9.29)	0.273	3.39 (-2.52, 9.30)	0.264
** Fungus**	3.67 (-7.62, 14.95)	0.526	3.63 (-7.80, 15.05)	0.536	1.19 (-10.43, 12.81)	0.841
Treatment failure						
**Early mNGS *vs* Late mNGS**	0.15 (0.05, 0.50)	0.002	0.16 (0.05, 0.53)	0.003	0.17 (0.05, 0.66)	0.010
Presence of the following pathogens						
** *Mycobacterium tuberculosis***	0.49 (0.06, 4.10)	0.508	0.49 (0.06, 4.22)	0.513	0.59 (0.07, 5.41)	0.644
** Gram-negative**	3.23 (1.02, 10.26)	0.047	3.31 (1.01, 10.77)	0.047	5.21 (1.39, 19.43)	0.014
** Enterobacteriaceaeǂ**	5.60 (1.26, 24.87)	0.024	4.62 (1.01, 21.20)	0.049	5.71 (1.17, 28.03)	0.032
** Gram-positive**	1.61 (0.51, 5.11)	0.421	2.00 (0.59, 6.79)	0.264	3.07 (0.82, 11.50)	0.095
** *Staphylococcus aureus***	4.13 (1.15, 14.87)	0.030	5.25 (1.35, 20.38)	0.017	8.64 (1.95, 38.34)	0.005
** Streptococcusƚ**	0.56 (0.07, 4.73)	0.591	0.60 (0.07, 5.36)	0.648	0.78 (0.08, 7.30)	0.830
** Anaerobes**	0.56 (0.07, 4.73)	0.591	0.66 (0.07, 6.02)	0.714	0.91 (0.10, 8.74)	0.936
** Virus**	2.27 (0.82, 6.24)	0.113	2.53 (0.87, 7.36)	0.089	2.99 (0.95, 9.40)	0.061
** Fungus**	0.64 (0.07, 5.56)	0.688	0.47 (0.05, 4.28)	0.505	0.21 (0.01, 2.91)	0.242
Clinical resolution						
**Early mNGS *vs* Late mNGS**	2.99 (1.34, 6.65)	0.007	2.97 (1.32, 6.68)	0.009	3.03 (1.24, 7.40)	0.015
Presence of the following pathogens						
** *Mycobacterium tuberculosis***	0.11 (0.01, 0.89)	0.039	0.10 (0.01, 0.84)	0.034	0.08 (0.01, 0.68)	0.021
** Gram-negative**	1.02 (0.36, 2.87)	0.976	1.02 (0.36, 2.90)	0.967	1.08 (0.36, 3.23)	0.888
** Enterobacteriaceaeǂ**	1.15 (0.27, 4.87)	0.847	1.26 (0.29, 5.45)	0.761	1.43 (0.31, 6.55)	0.648
** Gram-positive**	1.68 (0.64, 4.41)	0.289	1.65 (0.62, 4.40)	0.315	1.41 (0.52, 3.81)	0.497
** *Staphylococcus aureus***	1.16 (0.35, 3.85)	0.810	1.13 (0.34, 3.77)	0.847	0.93 (0.28, 3.17)	0.913
** Streptococcusƚ**	1.47 (0.37, 5.81)	0.581	1.47 (0.37, 5.90)	0.587	1.34 (0.33, 5.44)	0.685
** Anaerobes**	2.45 (0.58, 10.38)	0.222	2.46 (0.56, 10.78)	0.231	2.27 (0.51, 10.13)	0.282
** Virus**	1.72 (0.76, 3.90)	0.197	1.76 (0.76, 4.10)	0.188	1.82 (0.76, 4.35)	0.179
** Fungus**	2.00 (0.45, 8.83)	0.360	2.24 (0.49, 10.16)	0.298	3.98 (0.67, 23.55)	0.128

#Adjust for age and gender; *Early mNGS *vs* Late mNGS, adjust for gender, age, hypertension, diabetes, cardiovascular diseases, pulmonary diseases, chronic kidney disease stage 4-5, cancer; Presence of the following pathogens, adjust for: gender, age, diabetes, immunosuppression; ǂEnterobacteriaceae group included *Escherichia coli and Klebsiella pneumoniae;* ƚ Streptococcus group included *Streptococcus pneumoniae, Peptostreptococcus stomatis and Streptococcus constellatus.*

### Associations between microbiota patterns and clinical outcomes

After adjusting for age, sex and major comorbidities, we found that the presence of gram-negative bacteria was associated with a significantly longer length of hospital stay (β 8.81, 95% CI 0.92–16.70; P=0.03). Among gram-negative bacteria, the impact of the presence of Enterobacteriaceae was greater, with a mean difference of 15.06 days (95% CI 4.64–25.49, P=0.006). In addition, the presence of gram-negative bacteria (OR 5.21, 95% CI 1.39–19.43, P=0.01), Enterobacteriaceae (OR 5.71, 95% CI 1.17–28.03, P=0.03) and *Staphylococcus aureus* (OR 8.64, 95% CI 1.95–38.34, P=0.005) was associated with an increased risk of treatment failure in patients with the absence of the above pathogens, considering the confounding effects of age, sex, diabetes and immunocompromised conditions. In addition, the presence of *Mycobacterium tuberculosis* was associated with a significantly lower probability of clinical resolution (OR 0.08, 95% CI 0.01–0.68; P=0.02). No significant associations were detected between the presence of any type of pathogen and overall survival. The detailed results of the Cox regression analysis of overall survival are shown in **Table 4**.

**Table 4:** Association between early mNGS timing, microbiota profile and overall survival.

Exposure	Non-adjusted		Adjust model I#		Adjust model II*	
	HR (95%CI)	P value	HR (95%CI)	P value	HR (95%CI)	P value
**Early mNGS *vs* Late mNGS**	0.18 (0.04, 0.84)	0.029	0.18 (0.04, 0.85)	0.031	0.18 (0.04, 0.94)	0.042
Presence of the following pathogens						
** *Mycobacterium tuberculosis***	NA		NA		NA	
** Gram-negative**	0.32 (0.04, 2.54)	0.281	0.31 (0.04, 2.58)	0.279	0.30 (0.03, 2.77)	0.291
** Enterobacteriaceaeǂ**	NA		NA		NA	
** Gram-positive**	0.72 (0.15, 3.35)	0.675	0.76 (0.15, 3.74)	0.736	0.80 (0.15, 4.34)	0.795
** *Staphylococcus aureus***	1.40 (0.30, 6.51)	0.672	1.55 (0.31, 7.67)	0.589	1.34 (0.22, 8.25)	0.750
** Streptococcusƚ**	NA		NA		NA	
** Anaerobes**	0.69 (0.09, 5.61)	0.731	0.72 (0.08, 6.76)	0.774	0.56 (0.05, 6.14)	0.634
** Virus**	2.10 (0.64, 6.92)	0.221	2.12 (0.64, 7.02)	0.219	1.96 (0.54, 7.13)	0.306
** Fungus**	NA		NA		NA	

#Adjust for age and gender;*Early mNGS *vs* Late mNGS, adjust for gender, age, hypertension, diabetes, cardiovascular diseases, pulmonary diseases, chronic kidney disease stage 4-5, cancer; Presence of the following pathogens, adjust for: gender, age, diabetes, immunosuppression; ǂEnterobacteriaceae included *Escherichia coli and Klebsiella pneumoniae;* ƚ Streptococcus group included *Streptococcus pneumoniae, Peptostreptococcus stomatis and Streptococcus constellatus.*

## Discussion

In the present study, we explored the diagnostic and prognostic value of mNGS in biopsied tissues from complex infections, investigated the total microbiome from different types of biopsied tissues, and correlated microbiota patterns with clinical outcomes. Our findings suggested that mNGS showed high sensitivity and specificity in diagnosing all types of pathogenic microorganisms from biopsied tissues, especially viruses, fungi and parasites, which are usually difficult to detect via conventional methods. In addition, we found that early mNGS sampling within 7 days after admission was associated with a significantly reduced length of hospital stay, improved overall survival and clinical resolution, and a decreased risk of treatment failure. The above results further emphasized the importance of mNGS in the overall process of managing infectious diseases, from enhancing the diagnosis to improving the prognosis.

The realm of clinical microbiology comprises two main areas: diagnostic microbiology, which involves identifying pathogens from clinical samples to inform patient management and treatment, and public health microbiology, which is dedicated to monitoring and tracking infectious disease outbreaks within communities. Traditional diagnostic methods in microbiology labs involve cultivating and isolating microorganisms, detecting specific pathogen antibodies through serology, and identifying microbial nucleic acids (either DNA or RNA) via PCR. Unlike most molecular tests that pinpoint a narrow range of pathogens via designated primers or probes, metagenomic techniques capture all DNA or RNA in a sample (Gu et al., 2019). This allows for a comprehensive examination of the entire microbiome, as well as the analysis of the patient’s genome or transcriptome (Chiu and Miller, 2019).

To date, several studies have highlighted the potential of mNGS in both clinical and public health contexts. For example, mNGS helped diagnose neuroleptospirosis in a critically ill 14-year-old patient, revealing the first instance where mNGS offered actionable clinical insights (Wilson et al., 2014). This accurate diagnosis led to effective antibiotic treatment and patient recovery. In public health, mNGS has been used to track *Escherichia coli* strain O104:H4 outbreaks and monitor antibiotic resistance in food through bacterial genome sequencing (Loman et al., 2013). Big data from mNGS are increasingly used for clinical applications, such as detecting antibiotic resistance from samples or analyzing human response data to predict infections and assess disease risk (Stefan et al., 2016; Gliddon et al., 2018; Langelier et al., 2018). Consequently, mNGS is shaping the future of precision diagnosis in infectious diseases, pushing personalized patient care forward.

In terms of the microbiota profile from biopsied infected tissue, nearly half of the infection samples were polymicrobial. The microbiota patterns differ across various types of biopsied tissues, which could guide clinicians in the prescription of empirical antibiotics before precise diagnosis. In our study, Staphylococcus and Mycobacterium were highly abundant among all bacteria in the skeletal articular system, whereas anaerobic bacteria were most common in biopsied tissue from the lung or mediastinum. The distribution of bacterial abundance in vertebrae and joints was similar to that reported in previous studies (Duarte and Vaccaro, 2013; Principi and Esposito, 2016; Babic and Simpfendorfer, 2017). Previously, tuberculosis was the primary cause of spinal infections. However, with advancements in the diagnosis and treatment of lung tuberculosis over the past half-century, its prevalence has significantly declined. According to current evidence, most spinal infections are caused by monomicrobial infection with *Staphylococcus aureus*, accounting for an incidence rate between 30% and 80% (Hadjipavlou et al., 2000; Petitjean et al., 2004). In addition, we identified other bacteria, including Enterobacteriaceae, anaerobic bacteria, and Streptococcus. In certain series, gram-negative bacteria such as *Escherichia coli* can constitute up to 25% of all spinal infections (Sobottke et al., 2008). Anaerobic agents are more commonly discovered in penetrating spine trauma (Lim et al., 2006). Owing to the limited sample size, the bacterial pattern of certain types of biopsied tissue needs to be expanded in future studies, especially in the brain, bone marrow and semen.

With respect to the virus pattern in biopsied tissue, Torque teno virus (TTV) was the most prevalent virus, especially in the bone marrow, brain, and skeletal articular system. TTV is a small, single-stranded DNA virus that is ubiquitously detected in human populations. There is no confirmed disease or pathology directly attributed to TTV in humans. However, the virus has been detected in both healthy individuals and those with various diseases, which makes its clinical relevance uncertain. Some studies have proposed the use of the TTV load as a biomarker (Jaksch et al., 2018; Pradier et al., 2020). For example, in the context of transplant patients, a high TTV load in the blood might be indicative of a state of immunosuppression (Jaksch et al., 2018; Grenda, 2021). This has led to suggestions that monitoring TTV levels could guide the adjustment of immunosuppressive therapy in transplant recipients, although this application is still under investigation. Additionally, some studies have proposed associations between TTV and certain diseases, including liver diseases and respiratory conditions (Naoumov et al., 1998; Ohbayashi et al., 2001; Maggi et al., 2003; Reshetnyak et al., 2020; Bal et al., 2022; Focosi et al., 2023). However, causality has not been established, and more research is needed to determine any direct link.

Several limitations of our study need to be addressed. First, our study was a retrospective cohort study, which was intrinsically subject to recall bias and selection bias. Nevertheless, we conducted multivariate regression analysis to adjust for sex, age and major comorbidities, hoping to minimize the confounding effect on clinical outcomes. Second, the sample size of each type of biopsied tissue was limited, and current microbiota profiles in certain tissues are more similar to a rudiment that needs to be further expanded upon in future studies. Third, the lack of RNA sequencing restricted our ability to detect RNA viruses; thus, only DNA virus infection was covered in our study. Fourth, the identification of antimicrobial resistance genes by mNGS was not performed in the reported cohort, which limited further discussion of antimicrobial resistance patterns of different micro-organisms from tissue biopsy in complex infection.

## Conclusion

In summary, the application of mNGS in tissue biopsy not only aids in accurately identifying infectious pathogens but also in tailoring treatment and improving therapeutic responses, ensuring optimal patient care. Early mNGS sampling within 7 days after admission was associated with a significantly reduced length of stay in the hospital and improved overall survival and clinical resolution. The presence of gram-negative bacteria, Enterobacteriaceae and Staphylococcus in biopsied tissues was more likely associated with a higher probability of treatment failure, which favored more aggressive antibiotic strategies with a narrower spectrum. More high-quality studies are needed to explore and expand the microbiota profile of infections from biopsied brain, semen and bone marrow tissues.

## Data Availability

The original contributions presented in the study are included in the article/**Supplementary Material**. Further inquiries can be directed to the corresponding authors.

## Supplementary material

Supplementary Figure 1 Distribution and classification of all types of pathogens.

Supplementary Figure 2 The details of polymicrobial infection.
